# Systemic glucocorticoid prescriptions pattern and factors of inappropriate use in primary care institutions of Southwest China

**DOI:** 10.3389/fpubh.2022.952098

**Published:** 2022-09-12

**Authors:** Xiaobo Luo, Shitao Yu, Zhen Zeng, Xunrong Zhou, Yuxi Liu, Lei Wang, Jiaqi Hu, Yue Chang

**Affiliations:** ^1^School of Public Health, Guizhou Medical University, Guiyang, China; ^2^Guiyang Public Health Clinical Center, Guiyang, China; ^3^Second Affiliated Hospital of Guizhou University of Traditional Chinese Medicine, Guiyang, China; ^4^School of Humanities and Management, Institute for Health Law and Policy, Guangdong Medical University, Dongguan, China; ^5^Primary Health Department of Guizhou Provincial Health Commission, Guiyang, China; ^6^School of Medicine and Health Management, Guizhou Medical University, Guiyang, China

**Keywords:** systemic glucocorticoids, prescription patterns, appropriateness, inappropriate use, primary care institutions

## Abstract

**Background:**

Inappropriate use of glucocorticoids in primary care institutions is serious. It not only causes economic burden, but leads to many adverse reactions. The purpose of this study is to explore systemic glucocorticoid prescription pattern and factors of inappropriate use in primary care institutions.

**Methods:**

This is a retrospective study. Systemic glucocorticoids prescribed in 58 primary care institutions in Guizhou province of Southwest China in 2020 were selected from the Health Information System. All prescriptions were classified as appropriate or inappropriate use. Inappropriate use was classified into the following two categories: (a) Inappropriate indications; (b) Inappropriate selection of glucocorticoids. Multivariate analysis was used to explore the factors associated with inappropriate use of systemic glucocorticoids.

**Results:**

A total of 63,315 glucocorticoid prescriptions were included in the analysis. Diseases of the respiratory system (60.8%) and diseases of the skin and subcutaneous tissue (23.1%) were the most common indications for use. Injections (89.8%) predominated and dexamethasone (86.5%) was the most prescribed glucocorticoid. 68.2% of all prescriptions were inappropriate. Compared to physicians with a college degree, physicians with a junior college (OR: 1.12, 95% CI: 1.08–1.17) and technical secondary education (OR: 1.12, 95% CI:1.05–1.19) were more likely to prescribe glucocorticoids inappropriately as were attending physicians (OR: 1.12, 95% CI: 1.01–1.25) and resident physicians (OR: 1.31, 95% CI: 1.15–1.48) compared to associate chief physicians. The risk of inappropriate glucocorticoid use was highest in patients 65 years of age and older (OR: 6.00, 95% CI: 5.62–6.40). In contrast, prescriptions given by injection were more likely to be used inappropriately than those given orally (OR: 0.44, 95% CI: 0.41–0.46).

**Conclusion:**

Inappropriate use of systemic glucocorticoids without appropriate indications was extremely prominent in primary care institutions of Guizhou Province, especially in diseases of the respiratory system and among the elderly. The risk of inappropriate glucocorticoid use was highest in patients 65 years of age and older. It is important to note that physicians younger than 33, with more than 40 years of service, and attending or residents were more likely to inappropriately prescribe glucocorticoids.

## Introduction

Since 1950, systemic glucocorticoids have been widely used to treat various inflammatory and autoimmune diseases, such as asthma, chronic obstructive pulmonary disease, and rheumatoid arthritis (1, 2). Although systemic glucocorticoids (oral or injection) play a significant role in clinical treatment, their inappropriate use can induce adverse consequences such as adrenal insufficiency, osteoporosis, hypertension, Cushing's syndrome, and gastrointestinal bleeding, which could increase the burden on medical care and even endanger the patient's life (3–12). Therefore, systemic glucocorticoids should be used strictly according to their indication (13).

Over the past 20 years, prescriptions for oral glucocorticoids have risen by 34% in the United Kingdom (14). Among 113 patients surveyed in India, 88.4% of glucocorticoids were considered inappropriate, including imprecise diagnosis and incorrect indications by standard clinical and evidence-based practice guidelines (15). In France, oral glucocorticoid prescription rates are over 17%, yet most were inappropriately used (16). In the United States, systemic glucocorticoids were used in more than 11% of acute respiratory tract infections, yet guidelines failed to support this practice (17). Inappropriate use of glucocorticoids is also common in China (18, 19), and this phenomenon is more prominent in primary care institutions (20, 21). A previous study reported that glucocorticoids accounted for 63.5% of inappropriate prescriptions in 27 primary care institutions (22). In order to promote the rational use of glucocorticoids, the Chinese government promulgated the clinical use guidelines of glucocorticoids as early as 2011 (23), but the expected effects have not been achieved in primary care institutions (20, 21).

Previous studies have focused on oral glucocorticoid prescribing patterns in adults, including trends in the prevalence of oral glucocorticoids (14, 16, 24, 25), common indications for glucocorticoids therapy (14, 25, 26), and common types of glucocorticoids use (14, 27). A recent study reported characteristics of patients and their association with inappropriate use of systemic glucocorticoids in upper respiratory tract infections (28). Several studies (15, 29, 30) have also focused on the inappropriate use of systemic glucocorticoids in primary care institutions. Other studies have described prescribing patterns of topical glucocorticoids (31–35), and factors associated with their inappropriate use (36–39). However, few studies have comprehensively examined the prescription patterns and factors associated with inappropriate use of systemic glucocorticoid in primary care institutions.

Therefore, we conducted an in-depth analysis on the prescription of systemic glucocorticoids in primary care institutions in Southwest China. The objective of this study is to describe the prescription patterns and determine associated factors for the inappropriate use of systemic glucocorticoids. The influencing factors of inappropriate use of systemic glucocorticoids were analyzed from the perspective of physicians and patients.

## Materials and methods

This study was approved by the Ethics Committee of Guizhou Medical University (REC. 2021 Ethics Approval No. 249).

## Study setting

The retrospective study was conducted in Guizhou, one of the poorest provinces in Southwest China. In China, the public hospital system consists of three levels from top to bottom: tertiary hospitals, secondary hospitals, and primary care institutions. Primary care institutions include township health centers and community health service centers. Primary care institutions do not require high education and certificates. People who have a technical secondary school degree and pass professional physician training can become junior outpatient physicians. Out of 1,399 township public hospitals in Guizhou rural areas, there are 132 hospitals using the same health information system (HIS) that was developed by Guizhou Lianke Weixin Technology Co., LTD (LWTC) authorized by the Information Center of Guizhou Health Commission. Due to the high mobility of primary physicians, our inclusion criteria were: outpatient physicians who had been in the primary care institutions for the whole year of 2020 and had prescribed glucocorticoids. Eventually, 58 eligible primary care institutions were included in this study.

### Data retrieval process

Glucocorticoid prescriptions and demographic characteristics of the patients visiting outpatient departments in 2020 were collected in primary care institutions of Guizhou using the HIS. Personal details of the physicians were obtained from the Personnel Management Department of each primary care institution. According to the 10^th^ edition of the International Classification of Diseases (ICD-10) (40), the common related diagnoses were grouped into 5 diagnostic categories (Diseases of the respiratory system; Diseases of the skin and subcutaneous tissue; Diseases of the musculoskeletal system and connective tissue; Diseases of the digestive system; and Symptoms, signs and abnormal clinical and laboratory findings, not elsewhere classified). Systemic glucocorticoids are more likely to cause side effects than topical glucocorticoids (41). Therefore, we only included systemic glucocorticoids, excluding topical glucocorticoids prescriptions such as nasal inhalation and skin creams. Systemic glucocorticoids were divided into long-acting glucocorticoids such as dexamethasone and betamethasone, intermediate-acting glucocorticoids such as prednisolone, methylprednisolone and triamcinolone, and short-acting glucocorticoids such as hydrocortisone and cortisone (42).

### Categorization of appropriateness of glucocorticoids use

As there is no international standard clinical guidelines for glucocorticoids, we referred to the *Chinese Clinical Application Guidelines for glucocorticoids* (summary of S1 documents), *Chinese Ministry of Health Standards for Hospital Prescription Review and Management* (summary of S2 documents) and the articles of Liu et al. (42) and Yasir et al. (43). Based on the above guidelines and references, glucocorticoid prescriptions were divided into two broad categories: appropriate and inappropriate. Since HIS did not provide glucocorticoid doses, treatment duration nor patient's characteristics, we could only classify inappropriate use of all prescriptions into the following two categories. (a) Inappropriate indication, such as systemic glucocorticoids prescribed for the common cold, fever, and pain relief; and (b) Inappropriate selection of glucocorticoids, such as short-acting rather than long-acting systemic glucocorticoids.

### Statistical analysis

Appropriate and inappropriate prescribing frequencies were calculated to quantify the patterns and appropriateness of systemic glucocorticoid prescriptions. Univariate analysis was performed to initially exclude irrelevant factors of inappropriate use of systemic glucocorticoid. Multivariate analysis under the framework of Generalized Estimating Equations (GEE) was used to further determine independent predictors of inappropriate use of systemic glucocorticoids. All statistical tests were two-sided and a *p*-value <0.05 was considered statistically significant. R version 4.1.1 was used for all statistical analyses.

## Results

A total of 69,554 systemic glucocorticoid prescriptions were retrieved. Prescriptions given to patients with diagnoses in the top five diagnoses accounted for 91.0% of the total prescriptions. For this reason, we included only prescriptions given to these patients, resulting in 63,315 prescriptions in our analysis. The number of patients was 61,500.

Table 1 shows the distribution of systemic glucocorticoid prescriptions stratified by diagnosis, drugs and rationality. Out of the 63,315 prescriptions, 43,212 (68.2%) were used inappropriately, of which inappropriate indication prescriptions accounted for 67.7% and inappropriate selection of glucocorticoids prescriptions accounted for 0.5%. In the table, “A” represents “Appropriate use”, “I” represents “Inappropriate indications”, “S” represents “Inappropriate selection”. Diseases of the respiratory system (60.8%) was the most common diagnosis, followed by diseases of the skin and subcutaneous tissue (23.1%). Glucocorticoid prescriptions were inappropriate for 97.2% of respiratory diseases and 90.2% of digestive diseases.

**Table 1:** Prescribing patterns and appropriateness of systemic glucocorticoid prescriptions.

**Diagnosis**	**Total, *n* (%)**	**Injection (89.8)**					**Oral (10.2)**		**Appropriate use, *n* (%)**	**Inappropriate use**, ***n*** **(%)**	
		**Dexamethaso** **ne**	**Triamcinolo** **ne**	**Methylpredn** **isolone**	**Prednisolone**	**Hydrocorti** **sone**	**Dexametha** **sone**	**Prednisone**	**A**	**I**	**S**
**Diseases of the respiratory system**	**38,497 (60.8)**	**36,455 (66.6)**	**9 (39.1)**	**108 (72.5)**	**5 (3.9)**	**26 (1.4)**	**806 (20.6)**	**1,088 (43.4)**	**1,705 (2.8)**	**36,792 (97.2)**	**0 (0.0)**
**Acute upper respiratory infections**											
Acute upper respiratory infections of multiple and unspecified sites	18,521 (29.3)	**I** 17,610 (32.2)	**I** 14 (0.8)	**I** 35 (23.5)	**I** 4 (3.1)	**I** 4 (17.4)	**I** 383 (9.8)	**I** 471 (18.8)	0 (0.0)	18,521 (100.0)	0 (0.0)
Acute tonsillitis	5,551 (8.7)	**I** 5,478 (10)	**I** 1 (0.1)	**I** 16 (10.7)	0 (0.0)	**I** 3 (13.3)	**I** 12 (0.3)	**I** 41 (1.6)	0 (0.0)	5,551 (100.0)	0 (0.0)
Acute pharyngitis	1,107 (1.6)	**I** 1063 (1.9)	**I** 0 (0.0)	**I** 6 (4)	0 (0.0)	0 (0.0)	**I** 31 (0.8)	**I** 7 (0.3)	0 (0.0)	1107 (100.0)	0 (0.0)
Other diseases of upper respiratory tract	684 (1.1)	**I** 520 (0.9)	0 (0.0)	0 (0.0)	0 (0.0)	0 (0.0)	**I** 10 (0.3)	**I** 154 (6.1)	0 (0.0)	684 (100.0)	0 (0.0)
Chronic rhinitis, nasopharyngitis and pharyngitis	243 (0.3)	**I** 199 (0.4)	0 (0.0)	0 (0.0)	0 (0.0)	0 (0.0)	**I** 22 (0.6)	**I** 22 (0.9)	243 (100.0)	0 (0.0)	0 (0.0)
Acute laryngitis and tracheitis	192 (0.3)	**I** 157 (0.3)	0 (0.0)	0 (0.0)	0 (0.0)	0 (0.0)	**I** 33 (0.8)	**I** 2 (0.1)	192 (100.0)	0 (0.0)	0 (0.0)
**lower respiratory infection**											
Acute bronchitis	6,319 (10)	**I** 6,037 (11)	**I** 6 (0.3)	**I** 41 (27.5)	**I** 0 (0.0)	**I** 0 (0.0)	**I** 90 (2.3)	**I** 145 (5.8)	0 (0.0)	6,319 (100.0)	0 (0.0)
Bronchitis, not specified as acute or chronic	2,320 (3.7)	**I** 2,065 (3.8)	0 (0.0)	0 (0.0)	0 (0.0)	0 (0.0)	**I** 102 (2.6)	**I** 153 (6.1)	0 (0.0)	2,320 (100.0)	0 (0.0)
Other respiratory disorders	1,201 (1.8)	**A** 1,168 (2.1)	**A** 0 (0.0)	**A** 6 (4.0)	**A** 1 (0.1)	0 (0.0)	**A** 6 (0.2)	20 (0.8)	0 (0.0)	1,201 (100.0)	0 (0.0)
Unspecified chronic bronchitis	677 (1.1)	**A** 584 (1.1)	**A** 5 (0.3)	0 (0.0)	0 (0.0)	0 (0.0)	**A** 55 (1.4)	**A** 33 (1.3)	0 (0.0)	677 (100.0)	0 (0.0)
Pneumonia, organism unspecified	622 (1)	**A** 609 (1.1)	0 (0.0)	**A** 2 (1.3)	**A** 0 (0.0)	**A** 2 (8.7)	**A** 6 (2.2)	**A** 3 (0.1)	622 (100.0)	0 (0.0)	0 (0.0)
Asthma	444 (0.7)	**A** 407 (0.7)	0 (0.0)	**A** 1 (0.7)	0 (0.0)	0 (0.0)	**A** 25 (0.6)	**A** 11 (0.4)	444 (100.0)	0 (0.0)	0 (0.0)
Acute bronchiolitis	293 (0.5)	**A** 271 (0.5)	0 (0.0)	**A** 1 (0.7)	0 (0.0)	0 (0.0)	**A** 8 (0.2)	**A** 13 (0.5)	0 (0.0)	293 (100.0)	0 (0.0)
Other chronic obstructive pulmonary disease	204 (0.3)	**A** 176 (0.3)	0 (0.0)	0 (0.0)	0 (0.0)	0 (0.0)	**A** 18 (0.5)	**A** 10 (0.4)	204 (100.0)	0 (0.0)	0 (0.0)
Simple and mucopurulent chronic bronchitis	119 (0.2)	**I** 111 (0.2)	0 (0.0)	0 (0.0)	0 (0.0)	0 (0.0)	**I** 5 (0.1)	**I** 3 (0.1)	0 (0.0)	119 (100.0)	0 (0.0)
**Diseases of the skin and subcutaneous tissue**	**14,639 (23.1)**	**12,035 (22.1)**	**21 (1.2)**	**19 (12.8)**	**1 (0.8)**	**6 (26.1)**	**2,070 (49)**	**487 (19.4)**	**13,524 (92.4)**	**1,115 (7.6)**	**0 (0.0)**
Allergic contact dermatitis	11,074 (17.4)	**A** 8,978 (16.4)	**A** 15 (0.8)	**A** 8 (5.4)	**A** 1 (0.8)	**A** 1 (4.3)	**A** 1,748 (44.6)	3 **A** 23 (12.9)	11,074 (100.0)	0 (0.0)	0 (0.0)
Other local infections of skin and subcutaneous tissue	940 (1.5)	**I** 709 (1.3)	**I** 5 (0.3)	**I**	0 (0.0)	**I** 5 (21.7)	**I** 119 (5)	**I** 101 (4)	0 (0.0)	940 (100.0)	0 (0.0)
Urticaria	770 (1.1)	**A** 701 (1.3)	0 (0.0)	**A** 2 (1.3)	0 (0.0)	0 (0.0)	**A** 54 (1.4)	**A** 13 (0.5)	770 (100.0)	0 (0.0)	0 (0.0)
Atopic dermatitis	629 (1.0)	**A** 625 (1.1)	0 (0.0)	0 (0.0)	0 (0.0)	0 (0.0)	**A** 3 (0.1)	**A** 1 (0.0)	629 (100.0)	0 (0.0)	0 (0.0)
Other dermatitis	410 (0.6)	**A** 317 (0.6)	0 (0.0)	**A** 2 (1.3)	0 (0.0)	0 (0.0)	**A** 71 (1.8)	**A** 20 (0.8)	410 (100.0)	0 (0.0)	0 (0.0)
Irritant contact dermatitis	343 (0.5)	**A** 309 (0.6)	0 (0.0)	0 (0.0)	0 (0.0)	0 (0.0)	19 (0.5)	15 (0.6)	343 (100.0)	0 (0.0)	0 (0.0)
Pruritus	161 (0.3)	**A** 149 (0.3)	**A** 1 (0.1)	0 (0.0)	0 (0.0)	0 (0.0)	**A** 10 (0.3)	**A** 1 (0.0)	161 (100.0)	0 (0.0)	0 (0.0)
Unspecified contact dermatitis	96 (0.2)	**A** 83 (0.2)	0 (0.0)	0 (0.0)	0 (0.0)	0 (0.0)	**A** 7 (0.2)	**A** 6 (0.2)	96 (100.0)	0 (0.0)	0 (0.0)
Acute lymphadenitis	72 (0.1)	**I** 55 (0.1)	0 (0.0)	**I** 6 (4.0)	0 (0.0)	0 (0.0)	**I** 10 (0.3)	**I** 1 (0.0)	0 (0.0)	72 (100.0)	0 (0.0)
Cellulitis	44 (0.1)	**I** 35 (0.1)	0 (0.0)	0 (0.0)	0 (0.0)	0 (0.0)	**I** 6 (0.2)	**I** 3 (0.1)	0 (0.0)	44 (100.0)	0 (0.0)
Other epidermal thickening	43 (0.1)	**I** 24 (0.0)	0 (0.0)	0 (0.0)	0 (0.0)	0 (0.0)	**I** 19 (0.5)	0 (0.0)	0 (0.0)	43 (100.0)	0 (0.0)
Dermatitis due to substances taken internally	21 (0.1)	**A** 14 (0.0)	0 (0.0)	0 (0.0)	0 (0.0)	0 (0.0)	**A** 6 (0.2)	**A** 1 (0.0)	21 (100.0)	0 (0.0)	0 (0.0)
Cutaneous abscess, furuncle and carbuncle	16 (0.1)	**A** 15 (0.0)	0 (0.0)	0 (0.0)	0 (0.0)	0 (0.0)	0 (0.0)	**A** 1 (0.0)	0 (0.0)	16 (100.0)	0 (0.0)
Lichen simplex chronicus and prurigo	13 (0.1)	**A** 13 (0.0)	0 (0.0)	0 (0.0)	0 (0.0)	0 (0.0)	0 (0.0)	0 (0.0)	13 (100.0)	0 (0.0)	0 (0.0)
Acne	7 (0.1)	**A** 6 (0.0)	0 (0.0)	0 (0.0)	0 (0.0)	0 (0.0)	0 (0.0)	**A** 1 (0.0)	7 (100.0)	0 (0.0)	0 (0.0)
**Diseases of the musculoskeletal system and connective tissue**	**5,632 (8.9)**	**2,836 (5.2)**	**1,733 (95.4)**	**112 (8.1)**	**118 (92.2)**	**8 (34.8)**	**457 (10.8)**	**468 (18.7)**	**4,514 (80.1)**	**1,096 (19.5)**	**22 (0.4)**
Other arthritis	2,396 (3.7)	**A** 1,357 (2.5)	**A** 545 (30)	**A** 5 (3.4)	2 **A** 6 (20.1)	**S** 5 (21.7)	**A** 307 (5.8)	**A** 151 (6)	2,391 (99.8)	0 (0.0)	5 (0.2)
Other joint disorders, not elsewhere classified	730 (1.2)	416 (0.8)	238 (13.1)	0 (0.0)	14 (10.9)	S 1 (4.3)	**A**	**A** 33 (1.3)	729 (99.9)	0 (0.0)	1 (0.1)
Gout	524 (0.8)	**A** 373 (0.7)	**A** 54 (3.0)	**A** 6 (4.0)	**A** 2 (0.0)	0 (0.0)	**A** 53 (1.4)	**A** 36 (1.4)	524 (100.0)	0 (0.0)	0 (0.0)
Other arthrosis	487 (0.8)	**A** 79 (0.1)	**A** 219 (12.1)	0 (0.0)	**A** 4 (3.1)	0 (0.0)	**A** 38 (1.0)	**A** 147 (5.9)	487 (100.0)	0 (0.0)	0 (0.0)
Spondylosis	359 (0.6)	**I** 139 (0.3)	**I** 173 (9.5)	0 (0.0)	**I** 3 (2.3)	0 (0.0)	**I** 3 (0.1)	**I** 41 (1.6)	0 (0.0)	359 (100.0)	0 (0.0)
Other intervertebral disc disorders	298 (0.5)	**I** 75 (0.1)	**I** 205 (11.3)	0 (0.0)	**I** 7 (5.5)	0 (0.0)	**I**	**I** 9 (0.4)	0 (0.0)	298 (100.0)	0 (0.0)
Dorsalgia	253 (0.4)	**I** 140 (0.3)	**I** 84 (4.6)	0 (0.0)	**I** 7 (5.5)	0 (0.0)	**I** 9 (0.2)	**I** 13 (0.5)	0 (0.0)	253 (100.0)	0 (0.0)
Shoulder lesions	217 (0.3)	**A** 57 (0.1)	**A** 131 (7.2)	**A** 1 (0.7)	**A** 11 (8.6)	0 (0.0)	**A** 5 (0.1)	**S** 12 (0.5)	205 (94.5)	0 (0.0)	12 (5.5)
Other soft tissue disorders, not elsewhere classified	186 (0.3)	**I** 151 (0.3)	**I** 1 (0.1)	0 (0.0)	**I** 3 (2.3)	0 (0.0)	**I** 7 (0.2)	**I** 24 (1.0)	0 (0.0)	186 (100.0)	0 (0.0)
Synovitis and tenosynovitis	182 (0.3)	**A** 49 (0.1)	**A** 83 (4.6)	0 (0.0)	**A** 41 (32.0)	**S** 2 (8.7)	**A** 5 (2.1)	**S** 2 (2.9)	178 (97.8)	0 (0.0)	4 (2.2)
**Diseases of the digestive system**	**3,672 (5.8)**	**2,712 (5.0)**	**27 (1.5)**	**10 (6.7)**	**3 (2.3)**	0 (0.0)	**478 (11.3)**	**442 (17.6)**	**360 (9.8)**	**3,312 (90.2)**	**0 (0.0)**
Gingivitis and periodontal diseases	1,895 (3.0)	**A** 1,278 (2.3)	**A** 3 (0.2)	3 (2.0)	0 (0.0)	0 (0.0)	**A** 308 (7.9)	**A** 303 (12.1)	0 (0.0)	1,895 (100.0)	0 (0.0)
Diseases of pulp and periapical tissues	396 (0.6)	**I** 346 (0.6)	0 (0.0)	**I** 1 (0.7)	0 (0.0)	0 (0.0)	**I** 37 (0.9)	**I** 12 (0.5)	0 (0.0)	396 (100.0)	0 (0.0)
Gastritis and duodenitis	381 (0.6)	**I** 285 (0.5)	**I** 20 (1.1)	**I** 4 (2.7)	**I** 2 (1.6)	0 (0.0)	**I** 60 (1.5)	**I** 10 (0.4)	0 (0.0)	381 (100.0)	0 (0.0)
Other noninfective gastroenteritis and colitis	360 (0.6)	**A** 337 (0.6)	**A** 0 (0.8)	**A** 2 (1.3)	**A** 1 (0.0)	0 (0.0)	**A** 15 (0.4)	**A** 5 (0.2)	360 (100.0)	0 (0.0)	0 (0.0)
Stomatitis and related lesions	336 (0.5)	**I** 236 (0.4)	0 (0.0)	0 (0.0)	0 (0.0)	0 (0.0)	**I** 19 (0.5)	**I** 81 (3.2)	0 (0.0)	336 (100.0)	0 (0.0)
Functional dyspepsia	95 (0.2)	85 (0.2)	0 (0.0)	0 (0.0)	0 (0.0)	0 (0.0)	6 (0.2)	4 (0.2)	0 (0.0)	95 (100.0)	0 (0.0)
Other disorders of teeth and supporting structures	83 (0.1)	**I** 37 (0.1)	0 (0.0)	0 (0.0)	0 (0.0)	0 (0.0)	**I** 21 (0.5)	**I** 25 (1)	0 (0.0)	83 (100.0)	0 (0.0)
Embedded and impacted teeth	67 (0.1)	**I** 67 (0.1)	0 (0.0)	0 (0.0)	0 (0.0)	0 (0.0)	0 (0.0)	0 (0.0)	0 (0.0)	67 (100.0)	0 (0.0)
Cholecystitis	31 (0.1)	**I** 21 (0.0)	**I** 3 (0.2)	0 (0.0)	0 (0.0)	0 (0.0)	**I** 5 (0.1)	**I** 2 (0.1)	0 (0.0)	31 (100.0)	0 (0.0)
Diseases of tongue	28 (0.1)	**I** 23 (0.0)	**I** 1 (0.1)	0 (0.0)	0 (0.0)	0 (0.0)	**I** 4 (0.1)	0 (0.0)	0 (0.0)	28 (100.0)	0 (0.0)
**Symptoms, signs and abnormal clinical and laboratory findings, not elsewhere classified**	**875 (1.4)**	**734 (1.4)**	**9 (0.5)**	**0 (0.0)**	**1 (0.8)**	**0 (0.0)**	**108 (2.5)**	**23 (0.9)**	**0 (0.0)**	**526 (60.1)**	**349 (39.9)**
Rash and other nonspecific skin eruption	349 (0.6)	**S** 256 (0.5)	0 (0.0)	0 (0.0)	0 (0.0)	0 (0.0)	S 92 (2.3)	S 1 (0.0)	0 (0.0)	0 (0.0)	349 (100.0)
Cough	91 (0.1)	**I** 84 (0.2)	0 (0.0)	0 (0.0)	0 (0.0)	0 (0.0)	**I** 3 (0.1)	**I** 4 (0.2)	0 (0.0)	91 (100.0)	0 (0.0)
Abdominal and pelvic pain	91 (0.1)	**I** 86 (0.2)	0 (0.0)	0 (0.0)	0 (0.0)	0 (0.0)	**I** 3 (0.1)	**I** 2 (0.1)	0 (0.0)	91 (100.0)	0 (0.0)
Fever of other and unknown origin	90 (0.1)	90 (0.2)	0 (0.0)	0 (0.0)	0 (0.0)	0 (0.0)	0 (0.0)	0 (0.0)	0 (0.0)	83 (100.0)	0 (0.0)
Dizziness and giddiness	83 (0.1)	**I** 77 (0.1)	**I** 3 (0.2)	0 (0.0)	0 (0.0)	0 (0.0)	**I** 1 (0.0)	**I** 2 (0.1)	0 (0.0)	90 (100.0)	0 (0.0)
Headache	63 (0.1)	**I** 52 (0.1)	**I** 2 (0.1)	0 (0.0)	0 (0.0)	0 (0.0)	**I** 6 (0.2)	**I** 3 (0.1)	0 (0.0)	63 (100.0)	0 (0.0)
Pain in throat and chest	50 (0.1)	**I** 46 (0.1)	**I** 4 (0.2)	0 (0.0)	0 (0.0)	0 (0.0)	0 (0.0)	0 (0.0)	0 (0.0)	50 (100.0)	0 (0.0)
Enlarged lymph nodes	27 (00.1)	**I** 21 (0.0)	0 (0.0)	0 (0.0)	0 (0.0)	0 (0.0)	**I** 1 (0.0)	**I** 5 (0.2)	0 (0.0)	27 (100.0)	0 (0.0)
Pain, not elsewhere classified	18 (0.1)	**I** 12 (0.0)	0 (0.0)	0 (0.0)	**I** 1 (0.8)	0 (0.0)	0 (0.0)	**I** 5 (0.2)	0 (0.0)	18 (100.0)	0 (0.0)
Oedema, not elsewhere classified	13 (0.1)	10 (0.1)	0 (0.0)	0 (0.0)	0 (0.0)	0 (0.0)	**I** 2 (0.1)	**I** 1 (0.0)	0 (0.0)	13 (100.0)	0 (0.0)
**Total, n (%)**	**63,315**	**54,773 (86.5)**	**1,816 (2.9)**	**149 (0.2)**	**128 (0.2)**	**23 (0.1)**	**3,918 (6.1)**	**2,508 (4.0)**	**20,103 (31.8)**	**42,841 (67.7)**	**0 (0.5)**

A, Appropriate use; I, Inappropriate indications; S, Inappropriate selection. The bold values indicate the number and frequency of prescriptions for each system disease to the reader.

There were 20,103 (31.8%) prescriptions for appropriate use of glucocorticoids. The main indications for appropriate use of systemic glucocorticoids were asthma (100%), other chronic obstructive pulmonary disease (100%), skin and subcutaneous tissue diseases (92.4%), and musculoskeletal system and connective tissue diseases (80.1%).

Injection and oral glucocorticoids accounted for 89.8% and 10.2% of all prescriptions, respectively. There were 5 types of glucocorticoids for injection and only 2 types for oral route. Dexamethasone (86.5% and 6.2%) was the most frequently used glucocorticoid for both injection and oral routes.

Table 2 compares prescription patterns by physicians' and patients' characteristics. The majority (76.4%) of prescriptions were covered by the new rural cooperative scheme. On univariate analysis, there were significant differences in prescribing patterns among physicians by sex, age group, education, professional title and work duration (*P* < 0.001). In addition to patients' sex (*P* = 0.162), the appropriateness of glucocorticoid prescription was also related to patients' age, source of payment and route of use (*P* < 0.001). Therefore, patients' sex was excluded in the initial multivariate model.

**Table 2:** Factors associated with inappropriate use of glucocorticoids on univariate analysis.

**Variables**	**Total, n (%)**	**Appropriate use, n (%)**	**Inappropriate use, n (%)**		
			**Inappropriate indications**	**Inappropriate selection**	**P-value**
	**63,315**	**20,103 (31.8)**	**42,841 (67.7)**	**371 (0.5)**	
**Physician characteristic**					
**Sex**					
Female	20,075 (31.7)	6,506 (32.4)	13,361 (66.6)	208 (1.0)	<0.001
Male	43,240 (68.3)	13,597 (31.4)	29,480 (68.2)	163 (0.4)	
**Age group (years)**					
24–32	24,430 (38.6)	7,558 (31.0)	16,618 (68.0)	254 (1.0)	<0.001
33–40	18,892 (29.8)	6,272 (33.2)	12,515 (66.2)	105 (0.6)	
41–64	19,993 (31.6)	6,273 (31.4)	13,708 (68.5)	12 (0.1)	
**Education**					
College	22,824 (36.0)	7,142 (31.3)	15,667 (68.6)	15 (0.1)	<0.001
Junior college	28,241 (44.0)	8,967 (31.8)	18,935 (67.0)	339 (1.2)	
Technical secondary school	12,250 (20.0)	3,994 (32.6)	8,239 (67.3)	17 (0.1)	
**Professional title**					
Associate chief physician	1,503 (2.4)	507 (33.7)	994 (66.2)	2 (0.1)	<0.001
Attending physician	6,791 (10.7)	2,231 (32.9)	4,560 (67.1)	0 (0.0)	
Resident physician	55,021 (86.9)	17,365 (31.6)	37,287 (67.7)	369 (0.7)	
**Work duration (years)**					
≤ 5	15,970 (25.2)	4,811 (30.1)	10,895 (68.2)	264 (1.7)	<0.001
6–10	21,593 (34.1)	6,946 (32.2)	14,555 (67.4)	92 (0.4)	
11–20	8,264 (13.0)	3,005 (36.4)	5,254 (63.5)	5 (0.1)	
21–30	1,090 (17.2)	3,449 (31.6)	7,448 (68.3)	5 (0.1)	
31–39	5,707 (9.1)	1,643 (28.8)	4,063 (71.1)	1 (0.1)	
≥40	879 (1.4)	249 (28.3)	626 (71.2)	4 (0.5)	
**Patient characteristics**					
**Sex**					
Female	30,704 (48.5)	9,641 (31.4)	20,887 (68.0)	176 (0.6)	0.162
Male	32,611 (51.5)	10,462 (32.1)	21,954 (67.3)	195 (0.6)	
**Age group (years)**					
<5	10,288 (16.3)	249 (12.7)	10,012 (87.1)	27 (0.2)	<0.001
5–17	12,354 (19.5)	3,533 (28.6)	8,753 (70.8)	68 (0.6)	
18–49	17,465 (27.2)	5,285 (30.3)	12,065 (69.0)	115 (0.7)	
50–64	10,917 (17.1)	4,307 (39.4)	6,546 (60.0)	64 (0.6)	
≥65	5,616 (8.9)	5,519 (49.8)	5,465 (49.3)	97 (0.9)	
**Routes of use**					
Injection	56,889 (89.8)	16,829 (29.6)	39,796 (70.0)	264 (0.5)	<0.001
Oral	6,426 (10.2)	3,274 (50.9)	3,045 (47.4)	107 (1.7)	
**Payment source**					
New rural cooperative medical system (NCMS)	4,873 (76.4)	15,704 (32.5)	32,325 (66.8)	344 (0.7)	<0.001
Out-of-pocket	14,942 (23.6)	4,399 (29.4)	10,516 (70.4)	27 (0.2)	

Table 3 shows factors associated with inappropriate glucocorticoids use on multivariate analysis. Physicians who were male or older than 32 years (compared to those aged less than or equal to 32 years) were less likely to prescribe glucocorticoids inappropriately. Physicians with a junior level of education (compared to those who completed a college degree) and attending/resident physicians (compared to associate chief physicians) were more likely to prescribe glucocorticoids inappropriately. Compared to physicians with 5 or fewer years of work duration, those who had worked for 11–39 years were less likely to prescribe glucocorticoids inappropriately. However, physicians with 40 or more years of service were more likely to prescribe glucocorticoids inappropriately. From the patient's perspective, those who were five years of age or older (compared to those younger than 5 years) were more likely to be prescribed glucocorticoids inappropriately and the odds was the highest in patients aged 65 years or more. Injection prescriptions and prescriptions covered by the new rural cooperative medical system had a higher odds of inappropriate use.

**Table 3:** Factors predicting inappropriate use of systemic glucocorticoids on multivariate analysis.

**Variables**	**Adjusted OR (95% CI)**	***P*-value**
**Physician characteristics**		
**Sex**		
Female	**Ref**	
Male	0.92 (0.89–0.96)	<0.001
**Age group (years)**		
24–32	**Ref**	
33–40	0.87 (0.82–0.91)	<0.001
41–64	0.71 (0.64–0.78)	<0.001
**Education**		
College	Ref	
Junior college	1.12 (1.08–1.17)	<0.001
Technical secondary school	1.12 (1.05-1.19)	<0.001
**Professional title**		
Associate chief physician	**Ref**	
Attending physician	1.12 (1.01–1.25)	0.049
Resident physician	1.31 (1.15–1.48)	<0.001
**Work duration (years)**		
≤ 5	**Ref**	
6–10	0.98 (0.92–1.02)	0.390
11–20	0.60 (0.55–0.64)	<0.001
21–30	0.69 (0.61–0.76)	<0.001
31–39	0.88 (0.77–0.98)	0.033
≥40	1.59 (1.31–1.92)	<0.001
**Patient characteristics**		
**Age group (years)**		
<5	**Ref**	
5–17	2.61 (2.44–2.78)	<0.001
18–49	2.74 (2.58–2.91)	<0.001
50–64	3.93 (3.72–4.24)	<0.001
≥65	6.00 (5.62–6.40)	<0.001
**Routes of use**		
Injection	**Ref**	
Oral	0.44 (0.41–0.46)	<0.001
**Payment source**		
New rural cooperative medical system (NCMS)	**Ref**	
Out-of-pocket	0.96 (0.91–0.99)	<0.04

## Discussion

In this retrospective study, we analyzed 63,315 systemic glucocorticoid prescription patterns in 58 primary care institutions in Guizhou, China. Inappropriate use accounted for 68.2% of all glucocorticoid prescriptions, in which inappropriate indications was the majority (67.7%), and inappropriate selection of glucocorticoids accounted for 0.5%. Diseases of the respiratory system (60.8%) and diseases of the skin and subcutaneous tissue (23.1%) were the most common diagnoses among the patients who received the prescriptions. The proportion of inappropriate prescriptions was higher in diseases of the respiratory system (97.2%) and diseases of the digestive system (90.2%). Most (89.8%) glucocorticoid prescriptions were injected with only 10.2% given orally. Dexamethasone was the most used in both oral (6.2%) and injectable forms (86.5%). The younger physicians were more likely to prescribe inappropriately and the risk of inappropriate prescription was higher for physicians with lower educational and professional titles.

Among all patients prescribed systemic glucocorticoids in this study, prescriptions for diseases of the respiratory system (60.8%) were the most common, of which 97.2% of the prescriptions were inappropriate. A study from Ethiopia (27) showed glucocorticoids were used in 63.5% of respiratory diseases. Another study from China also reported that 60.48% of glucocorticoids were used for respiratory diseases (44). In Puerto Rico, 75% patients with of common colds were prescribed corticosteroids (45). Studies from the US and South Korea reported that 11% and 6.8% of systemic glucocorticoids were used for acute upper respiratory tract infections, respectively (28, 46). However, evidence-based clinical practice guidelines and Centers for Disease Control and Prevention (CDC) (47–51) suggest that systemic glucocorticoids are ineffective in acute respiratory tract infections since respiratory tract infections are usually caused by viruses such as acute bronchitis and acute tonsillitis. These conditions are self-healing with symptomatic treatment.

A previous study of digestive diseases showed an inappropriate rate of 69.0% (52). In this study, inappropriate use of systemic glucocorticoids was also common in diseases of the digestive system (90.2%). The largest number of prescriptions were for gingivitis and periodontal diseases. The main reason for using glucocorticoids was to relieve the patient's pain and reduce swelling during acute attacks. However, gingivitis is caused by the accumulation of substances produced by microbial plaque in or near the gum groove (53). Periodontitis may be associated with bacteria and the herpes viruses (54). Studies have shown that non–operative treatment for gingivitis and periodontitis should begin with special mechanical removal of plaque and calculus. Adjuvant therapy with antibiotics may also be used, but glucocorticoids are not recommended (55–58). In addition, a review study indicated that glucocorticoid use may increase the incidence of periodontitis (58).

In this study, systemic glucocorticoids used for COPD (204, 0.3%), asthma (444, 0.7%), and arthritis other arthritis (2,396, 3.7%), other joint disorders, not elsewhere classified (730,1.2%), other arthrosis (487,0.8 %), respectively. We only analyzed systemic glucocorticoid prescriptions, excluding topical glucocorticoid prescriptions. All diseases are classified according to the first three codes of ICD-10. In fact, these prescriptions for “Other Chronic obstructive pulmonary disease (J44)” contained only “acute exacerbation of undesignated COPD (J44.155) in the HIS”. Short-term, systemic glucocorticoids were prescribed to relieve symptoms. In addition, primary care physicians reported that systemic glucocorticoids are often used in conjunction with bisphosphonates in rheumatoid arthritis or osteoarthritis disease. The STOPP-START criteria mention that moderate to severe asthma or chronic obstructive pulmonary disease should be treated with regular inhaled glucocorticoids rather than long-term systemic glucocorticoids. And long-term use of glucocorticoids for rheumatoid arthritis or osteoarthritis should also be concomitant with bisphosphonates (59). And previous studies have shown that short-term systemic glucocorticoids could be used for remission in severe or acute asthma (60, 61), COPD (62), and arthritis (63, 64). Therefore, we comprehensively considered that short-term use of systemic glucocorticoids in severe COPD and rheumatoid arthritis was appropriate in this study.

Up to 68.2% of systemic glucocorticoids were used inappropriately in this study. A study in China found that 56.55% of glucocorticoid prescriptions were inappropriate (65). Similarly, Masih et al. (15) also reported that 88.4% of glucocorticoids were inappropriately used, which was a common phenomenon in rural areas of India. Due to the difficulty of finding the optimal induction and maintenance doses, glucocorticoids were also commonly misused in systemic lupus erythematosus in Spain (66). A United States study (30) reported that primary physicians often prescribed systemic corticosteroids for no apparent reason. Among the inappropriate prescriptions, 67.7% of prescriptions were inappropriate indications and 0.5% of prescriptions were inappropriate selection of glucocorticoids in this study. This suggests that most primary physicians may not have sufficient knowledge of the indications for glucocorticoids and individual physicians may have poor understanding of the characteristics of glucocorticoid drugs.

Injectable of glucocorticoids (89.8%) was more common than oral glucocorticoids in this study. In China (67), the proportion of glucocorticoids used for injection reached 71.42% while in Serbian (27) 52.6% of systemic glucocorticoid prescriptions were injections. A study reported that 22.5% of acute respiratory infections were treated with intramuscular glucocorticoids in the United States (68). Overuse of injected glucocorticoids not only increases medical costs, but also increases the transmission of iatrogenic diseases and the probability of side effects (21). Generally, systemic glucocorticoids are used in patients with exacerbations or emergencies. However, there is no advanced equipment to assist in the examination or rescuing patients in primary care institutions (69). Once suffering from acute or serious illness, most outpatients will choose to go to higher level health care institutions. Therefore, they are mild in primary care institutions in China. In general, injection glucocorticoids are not necessary in primary care institutions (70, 71). In this study, dexamethasone was the most prescribed glucocorticoid. Li Jia et al. (67) also reported a high prevalence (90.92%) of dexamethasone use in China. This is similar to a study by Masih (15) where dexamethasone (58.3%) is also the most commonly prescribed drug in India.

In our study, physicians younger than 33 years and attending or resident physicians were more likely to prescribe glucocorticoids inappropriately. Most of these physicians are non-undergraduates and their professional knowledge and clinical experience are also inadequate. Physicians with more than 40 years of service were also more likely to prescribe glucocorticoids inappropriately. It has been reported that most elderly physicians in rural China have no formal education (72). In China, most young and middle-aged primary physicians still lack higher education (73). Xu et al. (72) recommended that better welfare policies be set up to attract more highly educated physicians to the countryside while Li et al. (74) suggested that remote education and clinical continuing education could be used to train rural physicians to improve the professional knowledge of physicians.

Inappropriate use of systemic glucocorticoids was also associated with patient's age and route of use in this study. The inappropriate use of glucocorticoids became more pronounced with the patient's age, especially those 65 years and older. In rural China, most elderly citizens lack formal education (75). Education level is positively correlated with health literacy (76, 77). Yuan et al. (78) also reported that rural residents over 65 years old had the lowest rate of health knowledge. In our study, injection glucocorticoids were more likely to be used inappropriately than those given orally. Indeed, it is still widely believed that injections are more convenient and effective than oral drugs (21). Because few people are aware of the harmful consequences of inappropriate use of injections, it is common for patients to request injectable treatment to quickly recover from their disease (79). In addition, glucocorticoid use covered by the New Rural Cooperative Medical scheme were more likely to be inappropriate than those paid out-of-pocket. In rural China, most people have rural health insurance, which reduces the cost of medical care and thus may increase patient's demand for drugs (80). Therefore, health education on the rational use of injections among the general population should be strengthened, and more targeted prescription medication policies and program management in primary care institutions should be formulated. It is also necessary to strengthen the public awareness of the potential risks of glucocorticoids to change traditional consumer attitudes. Dogba et al. (81) proposed the use of information and communication technologies to provide health education to the public in rural and remote areas.

Our study has some limitations. First, the appropriateness of glucocorticoids was classified based on incomplete retrospective data. So far as we know, the HIS of most primary care institutions in less developed areas of China are still being improved, and factors such as dose, treatment time, and patient's disease history are difficult to obtain. It is hoped that future studies will provide a more comprehensive classification of appropriateness. Secondly, as this study was conducted in only one province of China, the results may not be representative of systemic glucocorticoid use in all primary care institutions of China, its representation may not be sufficient. Even so, the study still can be replicated in areas of countries like the study, the results of the study are consistent with those set out in the objectives and the size of the sample allows these objectives to be measured, reaching the final conclusions. Thirdly, there is no international clinical medication guidelines for glucocorticoids at present, so the results of our evaluation might be inconsistent with the medication practices of other countries. We therefore call for the formulation of more comprehensive guidelines for the clinical use of glucocorticoids to promote their rational use and reduce the occurrence of adverse side effects.

## Conclusion

The inappropriate use of systemic glucocorticoids is an urgent problem in primary care institutions of Guizhou Province. The use of systemic glucocorticoids without appropriate indications was extremely prominent, especially in diseases of the respiratory system. It is important to note that physicians younger than 33, with more than 40 years of service, and attending or residents were more likely to inappropriately prescribe glucocorticoids.

## Data availability statement

The original contributions presented in the study are included in the article/supplementary material, further inquiries can be directed to the corresponding authors.

## Author contributions

YC provided conceptualization and design for this study. LW provided support and assistance for data acquisition. Clinical pharmaceutical technical support was provided by SY and ZZ. YC, XL, and JH analyzed the data. YC and XL completed the manuscript. XZ and YL provided supervision and assistance for the whole research process. All authors played an important role and approved the submitted version.

## Funding

This study was supported by the Natural Science Foundation of Guizhou Province, Feedback intervention model of Gradient Boosting Decision Tree (GBDT) technology on glucocorticoid prescription control in primary care institutions Guizhou Science And Technology Foundation -ZK [2021] General 499. And this study was also funded by the Medical Economics and Management Research Center of Guizhou Medical University (GMUMEM2022-A05).

## Conflict of interest

The authors declare that the research was conducted in the absence of any commercial or financial relationships that could be construed as a potential conflict of interest.

## Publisher's note

All claims expressed in this article are solely those of the authors and do not necessarily represent those of their affiliated organizations, or those of the publisher, the editors and the reviewers. Any product that may be evaluated in this article, or claim that may be made by its manufacturer, is not guaranteed or endorsed by the publisher.
